# Global mobility and the break-up of human population isolates – neglected mechanisms in health, demographics, and anthropology

**DOI:** 10.3325/cmj.2015.56.324

**Published:** 2015-08

**Authors:** Ozren Polašek

**Affiliations:** Medical School, University of Split, Split, Croatia

Throughout the greatest part of our evolutionary history, we were living in small and isolated tribal communities (1). Such a set-up ensured better survival in harsh environments and created distinctive genetic structure of groups that maintained sufficient level of isolation from one another. The traces of such events are clearly visible even today. For example, we are able to discern genetic origin of individuals from small villages that have maintained their genetic distinctiveness due to geographical isolation (2).

In contrast, over the course of just a few latest human generations, we have experienced substantial development, marked by industrialization and environmental changes, increased mobility and improvements of health care, which have irrevocably altered our previous lifestyles. Some changes, such as increased height and longer lifespan, were commonly attributable to better living conditions and socioeconomic improvements, but occasionally such phenomena were also suggested to have genetic origins (3,4). However, if such effects would indeed be under genetic control, they would have to produce an effect over a very short time. A possible explanation was proposed by the genome-based estimates of homozygosity (5), which were mainly propelled by the corresponding studies in animals and plants that suggested better phenotypic outcomes in more heterozygous organisms. The methodological problems related to the development and use of the best genomic measure (6) were resolved by the use of runs of homozygosity, ie, stretches of homozygous blocks of DNA in an individual’s genome (7).

The major limitation of previous studies, which attempted to describe the effects of increased homozygosity, resided in substantial confounding effects, low statistical power, and lack of replication across various ethnic groups. All these problems were surpassed in a recent study by Joshi et al (8), which demonstrated the detrimental effects of longer stretches of runs of homozygosity on height and cognitive ability. This study suggested that offspring of first cousins would experience a height reduction of 1.2 cm and cognitive ability reduction that would correspond to 10 months less of education (8). Although such effect sizes might not seem strong, they were recorded across different ethnic groups and populations from different continents, thus suggesting that the results were replicable and were not affected by confounding. This is the first solid piece of evidence that points to better phenotypical properties of children of genetically distant parents.

This study (8) confirms previous suggestions of poor phenotypic outcomes as a result of inbreeding, and offers a much deeper insight into the consequences of mobility vs isolation (4). In practical terms – the breakdown of the traditional tribal structure through isolate break-up, admixture of modern populations, and increase in mobility has contributed to the global positive trends of increased height and IQ. This translates into the direct negation of any kind of racism and other types of exclusivism and suggests that better phenotypic outcomes can be expected in admixed populations. Although this may hold true, there are still large methodological problems that prevent proper conclusions, due to very prevalent differences in general socioeconomic and living conditions and quality of health care across countries. The future studies will also have to be based on sufficient sample sizes. For example, a study based on nearly 6000 participants did not show an association between runs of homozygosity and survival to old age (9), which is another trait showing a steady improvements over the past few human generations.

Isolate break-up and its effects will surely become a more widely accepted mechanism in population health, demography, and anthropology. The widespread availability of genomic information, as well large-scale resources and biobanks (10), will facilitate our understanding of genetic mechanisms in the development of modern populations. In this instance, the *Croatian Medical Journal* welcomes and supports high quality studies in population genetics and application of this knowledge for medical and forensic purposes (11-18), aiming to contribute to the overall understanding of how genetics and societal changes affect our health and illness.
